# Comment on the study Coronary Artery Bypass Surgery in Brazil: Analysis of the National Reality Through the Bypass Registry that was presented at the 46^th^ Congress of the Brazilian Society of Cardiovascular Surgery, Nova Lima, BH, Brazil, April 5 and 6, 2019

**DOI:** 10.21470/1678-9741-2019-0606

**Published:** 2019

**Authors:** Luís Alberto O. Dallan

**Affiliations:** 1 Instituto do Coração do Hospital das Clínicas da Faculdade de Medicina da Universidade de São Paulo (InCor-HCFMUSP), São Paulo, SP, Brazil.

This cross-sectional study started in 2015, including 2,292 patients undergoing coronary artery bypass grafting (CABG) and cataloged in the BYPASS registry, aiming to constitute an institutional database on CABG in Brazil. The main objective was to analyze the profile, risk factors, results and surgical strategy employed in these patients.

It is noteworthy the small number of patients included (2,292), which shows little adherence to the registry. In addition, we know that even with a voluntary presentation, more complex patients with very bulky data are more difficult to report. As a comparison term, DATASUS sources show that 226,629 isolated CABG surgeries were performed in a 10-year period in Brazil (Jan 2008 to Feb 2018), with or without cardiopulmonary bypass (CPB).

The results of the study^[1]^ presented by the authors demonstrate well our reality: 42.5% were diabetic and 71% had previous acute myocardial infarction. An important information that I consider excellent, but out of a reality for all the cases performed in Brazil, is that 32.9% of the patients went through the "heart time". Probably the discussion promoted between the clinician and the interventional cardiologist was complemented by the surgeon.

Most CABGs were performed with CPB (87%) and cardioplegic arrest (95.2%), and the mean number of vessels treated was three. Only 6.9% of the operated patients received bilateral internal thoracic grafts, which is in agreement with the international databases, although there is great regional variation.

I consider the observed mortality (2.8%) low, close to that reported by STS (about 2.3%). At InCor-São Paulo, in 2018, 595 patients underwent CABG. The overall mortality was 3.9%, but when only elective and isolated CABG were considered, this percentage fell to 1.9%.

I salute the authors for the importance and relevance of the topic addressed. We know that records like the one proposed allow us to create a database that helps us better understand the difficulties and improve the results.

I leave some questions to Dr. Rodrigo: How do you explain such low mortality? What is the actual adjusted score of these patients (STS, Euroscore) and the evaluation of the data quality in relation to the accuracy? That is, if they are moderate or high-risk patients, better still. Did the mortality occur within 30 days or was in-hospital?

I close once again emphasizing that quality improvement programs in surgical patient care have been positively increasing the results of CABG surgery.
