# Supplementary material for: Comment on the study Coronary Artery Bypass Surgery in Brazil: Analysis of the National Reality Through the Bypass Registry that was presented at the 46th Congress of the Brazilian Society of Cardiovascular Surgery, Nova Lima, BH, Brazil, April 5 and 6, 2019
Source: Braz J Cardiovasc Surg. 2019 Jul-Aug;34(4):504–6. doi: 10.21470/1678-9741-2019-0606 (PMC6713380; doi:10.21470/1678-9741-2019-0606)
Supplement: Related texts

# Reply: The BYPASS Registry: Coping with our forthcoming needs

**Authors:** Rodrigo Pereira Paez, Nelson Américo Hossne Junior, José Amalth do Espírito Santo, Otavio Berwanger, Renato Hideo Nakagawa Santos, Renato Abdala Karam Kalil, Fabio B. Jatene, Alexandre Biasi Cavalcanti, Alexandre Cabral Zilli, Luiz Carlos Bettiati Jr, Fernando Augusto Marinho dos Santos Figueira, Stephanie Steremberg Pires D’Azevedo, Marcelo José Ferreira Soares, Marcio Pimentel Fernandes, Roberto Vito Ardito, Renata Andrea Barberio Bogdan, Valquíria Pelisser Campagnucci, Diana Nakasako, Clarissa Garcia Rodrigues, Anilton Bezerra Rodrigues Junior, Marcelo Matos Cascudo, Fernando Antibas Atik, Elson Borges Lima, Vinicius José da Silva Nina, Renato Albuquerque Heluy, Lisandro Gonçalves Azeredo, Odilon Silva Henrique Junior, José Teles de Mendonça, Katharina Kelly de Oliveira Gama Silva, Marcelo Pandolfo, José Dantas de Lima Júnior, Renato Max Faria, Jonas Gonçalves dos Santos, Guilherme Henrique Biachi Coelho, Sergio Nunes Pereira, Roberta Senger, Enio Buffolo, Guido Marco Caputi, Juliana Aparecida Borges de Oliveira, Walter J. Gomes, BYPASS Registry Study Group

**Affiliations:** 1 Hospital São Paulo, Escola Paulista de Medicina da Universidade Federal de São Paulo, (EPM - UNIFESP), São Paulo, SP, Brazil.; 2 Instituto de Pesquisa do Hospital do Coração (IP - HCor), São Paulo, SP, Brazil.; 3 Instituto de Cardiologia do Rio Grande do Sul - Fundação Universitária de Cardiologia, Porto Alegre, RS, Brazil.; 4 Cardiovascular Surgery Division, Instituto do Coração do Hospital das Clínicas da Faculdade de Medicina da Universidade de São Paulo (InCor-HCFMUSP), São Paulo, SP, Brazil.; 5 Hospital de Caridade São Vicente de Paulo, Jundiaí, SP, Brazil.; 6 Instituto de Medicina Integral Professor Fernando Figueira (IMIP), Recife, PE, Brazil.; 7 Hospital de Base - FUNFARME e FAMERP, São José do Rio Preto, SP, Brazil.; 8 Instituto de Moléstias Cardiovasculares (IMC), São José do Rio Preto, SP, Brazil.; 9 Irmandade da Santa Casa de São Paulo (INCT-HPV/Faculdade de Ciências Médicas da Santa Casa de São Paulo), São Paulo, SP, Brazil; 10 Instituto do Coração de Natal, Natal, RN, Brazil.; 11 Instituto de Cardiologia do Distrito Federal, Brasília, DF, Brazil.; 12 Hospital Universitário da Universidade Federal do Maranhão (HU/UFMA), São Luís, MA, Brazil.; 13 Hospital Evangélico, Cachoeiro de Itapemirim, ES, Brazil.; 14 Hospital do Coração de Sergipe, Aracaju, SE, Brazil.; 15 Instituto de Cirurgia Cardiovascular (ICCV)/Hospital Nossa Senhora da Salete, Cascavel, PR, Brazil.; 16 Hospital Wilson Rosado, Mossoró, RN, Brazil.; 17 Hospital Bosque da Saúde, São Paulo, SP, Brazil.; 18 Hospital Universitário de Santa Maria, Santa Maria, RS, Brazil.; 19 Hospital do Coração (HCor), São Paulo, SP, Brazil.

Dear Editor

We thank the comments of Professor Dallan on our paper^[1]^. Comprehensive national registries on medical procedures are an established necessity, then the reason for existence of the Bypass project.

Much like other similar databases, the beginning was troubled with budget constraints and restrictive participation of dedicated centers. Nonetheless, the project is gaining traction with guidelines from medical societies categorically stating that active participation in national registries or approved surgical database is essential and a condition for delivery of high-quality cardiovascular care in myocardial revascularization and valvular heart disease^[2,3]^. Additionally, establish a tool for understanding the real figures for the cardiovascular surgery practice in our country and develop strategies for improvements in quality and excellence.

Addressing the pertinent inquiries, the relative low mortality is clearly related to the quality of the selected centers involved so far and perhaps to the individual characteristics of patients referred for this treatment in our mean. A further study to clarify this aspect is in the pipeline. Also, the recently reformed BYPASS’ datasheet for data collection has incorporated the EuroSCORE risk model, which is likely to provide valuable information in stratifying outcomes accordingly.

Of note, the number of patients inserted in the BYPASS database has doubled in the last two years, with the perception by the surgical teams that their data is protected, the possibility of scientific production and the prospects of implementing continuous improvement processes^[4]^.

## References

[1] Paez RP, Hossne Junior NA, Santo J, Berwanger O, Santos RHN, Kalil RAK (2019). Coronary artery bypass surgery in Brazil: analysis of the
national reality through the BYPASS registry. Braz J Cardiovasc Surg.

[2] Neumann FJ, Sousa-Uva M, Ahlsson A, Alfonso F, Banning AP, Benedetto U (2019). 2018 ESC/EACTS Guidelines on myocardial
revascularization. Eur Heart J.

[3] Nishimura RA, O’Gara PT, Bavaria JE, Brindis RG, Carroll JD, Kavinsky CJ (2019). 2019 AATS/ACC/ASE/SCAI/STS expert consensus systems of care
document: a proposal to optimize care for patients with valvular heart
disease: a joint report of the American association for thoracic
surgery, American college of cardiology, American society of
echocardiography, society for cardiovascular angiography and
interventions, and society of thoracic surgeons. Catheter Cardiovasc Interv.

[4] Gomes WJ, Kalil RA, Jatene FB (2016). Brazilian registry of cardiovascular surgery in adults fully
operational. Braz J Cardiovasc Surg.
